# Environmental impact of the barrage construction on groundwater

**DOI:** 10.1007/s10653-026-03232-6

**Published:** 2026-05-19

**Authors:** Sherif M. Aboueldahab, El-Montser M. Seleem, Ahmed M. Orabi, Salah A. M. Zeid, Mariam Metwaly, Mahmoud A. Abdelhafiz

**Affiliations:** 1 Geology Department, Faculty of Science, Al-Azhar University, Assiut, 71524 Egypt; 2 Environmental Quality Management, Egyptian Environmental Affairs Agency, Assiut, Egypt; 3 Conservation Department, Faculty of Archaeology, Zagazig University, Zagazig, 44519 Egypt

**Keywords:** Groundwater behavior, Agroecosystem, Barrage construction, Groundwater quality index, Human health risk

## Abstract

Groundwater is among the most vital sources of freshwater, providing the primary supply for human consumption and for agricultural and industrial use. Although the construction of barrages enhances resource management and alleviates droughts and floods amid escalating population growth, it affects groundwater quantity and quality. As the most significant multipurpose water project on the Nile River, the new Assiut Barrage is crucial to the country’s food self-sufficiency and to the livelihoods of nearly 6 million people. However, the environmental impacts of the new barrage on groundwater in the surrounding area are unclear. In this study, we monitored groundwater levels at 55 wells over 12 years, both before and during barrage construction, to clearly illustrate the barrage’s effects on groundwater levels. Moreover, groundwater quality and associated health risks (HRA) were evaluated. This research demonstrated that the presence of water in a barrage’s reservoir affects the groundwater level, particularly in the surrounding area. During the construction of the new barrage, groundwater levels declined in the upstream observation wells while increasing in the downstream districts. This fluctuation could be due to excessive groundwater withdrawals for irrigation in the upstream areas, while levels decrease in the downstream areas because of their urban rather than agricultural character. According to the groundwater quality index (GQI) model, 75% of upstream groundwater samples and 66.7% of downstream samples fall into the good category for irrigation. Our HRA findings indicated that both carcinogenic and non-carcinogenic risks from dermal exposure to heavy metals (HMs) across all studied groundwater wells are negligible. This study provides a clear picture of the impacts of barrages on groundwater, with implications for similar conditions worldwide, including arid and semi-arid regions.

**Supplementary Information:**

The online version contains supplementary material available at 10.1007/s10653-026-03232-6.

## Introduction

Rivers are vital for ecosystem connectivity and conservation and serve as key sources of drinking, irrigation, domestic, and industrial water, making them essential for human existence and advancement. The construction of dams is primarily intended to enhance resource management and alleviate droughts and floods amid escalating population growth, urbanization, industrialization, and agricultural expansion (Javadinejad et al., 2019; Ren et al., 2019; Zang et al., 2024). Although barrage construction stores and provides water for irrigation, household use, flood reduction, electricity generation, streamflow, runoff velocity, and groundwater recharge (Çelik, 2018; Poff et al., 2007; Sulistiyono et al., 2021), it can also have undesirable environmental effects, which have been thoroughly documented (Collier et al. 1996, Graf, 2006, Magilligan & Nislow 2005). As an artificial intervention that modifies flow and hydraulic head gradients, the construction of barrages affects groundwater levels (Oh et al., 2016). It has been noted that the barrages alter hydraulic flow by increasing water depths and decreasing velocities in areas with established backwater curves (Gray, 1992; Novak et al., 2007). Because barrage construction provides irrigation water during the dry seasons, groundwater use decreases, thereby raising groundwater levels (Çelik, 2018; Killian et al., 2019). The barrage's impact on groundwater is expected to occur between upstream and downstream areas and may also affect the quantity of water available to humanity.

The new Assiut barrages and hydroelectric power plant project on the Nile River, the world's longest river, is one of the most significant multipurpose water projects in Egypt, as it secures water to irrigate around 0.7 million ha of agricultural land. As the Nile had previously undergone significant damming to generate hydropower and regulate flow to optimize irrigation and transportation (Omran & Negm 2019), the government built the new barrage to ensure a long-term irrigation supply, generate hydropower, and improve conditions for both river and road traffic. The new Assiut barrages were constructed across the River Nile to replace the old ones, dating back to 1898–1903. The existing barrage is approximately 400 m from the old barrage and 545 km downstream of the Aswan High Dam. It consists of 8 bays with 17 m-wide gates and two sluiceways to evacuate emergency flood release. Powerhouses are 4 × 8-MW bulb turbines, along with a road bridge. Double chamber navigation, head Pond level: 48–50 m a.s.l. and operating head: 3 to 6.7 m. As Egypt faces one of the highest water budget deficits in Africa (Abotalib et al., 2023), monitoring groundwater, the most vital source of freshwater, during the construction of new barrages is essential.

The environmental impact of the barrage on groundwater quality was highlighted (Baxter, 1977; Brainwood et al., 2004; Parimala renganayaki & Elango 2014). As highlighted above, barrages influence river ecosystems and modify the hydraulic regime, thereby impacting water quality by altering the transport and decay of pollutants along the river. The stream’s sediment-carrying capacity is reduced, leading to upstream sediment deposition. Furthermore, during flood seasons, dam operations may elevate river flow velocity, thereby facilitating the conveyance, transformation, and resuspension of contaminants, which deteriorates downstream water quality (Luo et al., 2021; Mazumder, 2004; Nafchi et al., 2022). Thus, the pollutant load will impact water quality differently after construction. Abdelhafiz et al. (2021a) highlighted that the construction of dams on the Nile River results in a deterioration of water quality. Therefore, a robust understanding of groundwater quality and related health risks is needed.

Groundwater resources are among the most vital sources of freshwater, providing the main supply for human consumption and for agricultural and industrial use where surface water is scarce (Abdelhafiz et al., 2021b; Delgado et al., 2010). Intensive groundwater withdrawals for agricultural irrigation have led to significant declines in groundwater levels and streamflow, raising concerns about the future availability of groundwater resources and the effects of these withdrawals on streamflow (Barlow & Clark 2011, Barlow & Leake 2012, Killian et al., 2019). Similar to surface water, groundwater can also be vulnerable to various sources of contamination. Population growth and increasing demand for water resources for various uses lead to declines in groundwater quality (Yang et al., 2019). Additionally, increasing groundwater overconsumption, rapid urbanization and industrialization, over-application of chemical fertilizers and pesticides, and inadequate drainage systems are key human activities that lead to deteriorated water quality, posing hazards to society and affecting human health (Abdelhafiz et al., 2021a; Karunanidhi et al., 2021; Koffi et al., 2017; Marghade et al., 2015; Sahoo & Swain 2020). Recent research has largely assessed groundwater quality and pollutants, which are intrinsically linked to human health, using the groundwater quality index (GQI) in several global locations (Abbasnia et al., 2019; Bouderbala, 2017; Chaurasia et al., 2018; D et al., 2021; Mostafa et al., 2023; Nsabimana et al., 2021; Ravindra et al., 2023; Tiwari et al., 2018; Zeid et al., 2018). Heavy metals (HMs) can occur at levels several times higher than natural background concentrations, polluting water and posing severe threats to human and ecological health. Due to their high toxicity, propensity for bioaccumulation, and long residence times, chronic exposure to HMs has been associated with neurological, reproductive, hepatic, immunological, and carcinogenic consequences (Aslam et al., 2024 Gan et al., 2026, Hu et al., 2020, Hu et al., 2017, Rai et al., 2019, USEPA 2000, Zheng et al., 2021). The health risk assessment (HRA) model has been widely used to evaluate the potential risks posed by HMs to human health across multiple pathways (Gao et al., 2021, Sheng et al., 2023, Shomar & Rashkeev 2021, Wang et al., 2025, Wei et al., 2025). Accurate assessment of the health hazards posed by H Ms in groundwater is essential for effective pollution management and for safeguarding public health.

The barrages alter the groundwater table, affecting both urban and agricultural areas; however, it is unclear how the new barrage affects the groundwater levels, particularly in the surrounding area. To bridge this knowledge gap, a critical question should be addressed: How does barrage construction affect both groundwater quantity and quality? Therefore, the main objective of the current work is to assess the primary environmental impacts of constructing the new barrage on groundwater levels using a long‑term monitoring dataset. Besides, groundwater quality from the observation wells and the associated health risks were evaluated. This work provides a database and presents a precise scenario of the effects of barrage construction on groundwater for the sustainable development of society.

## Methodology

### Study area description and groundwater monitoring

**Fig. 1:**
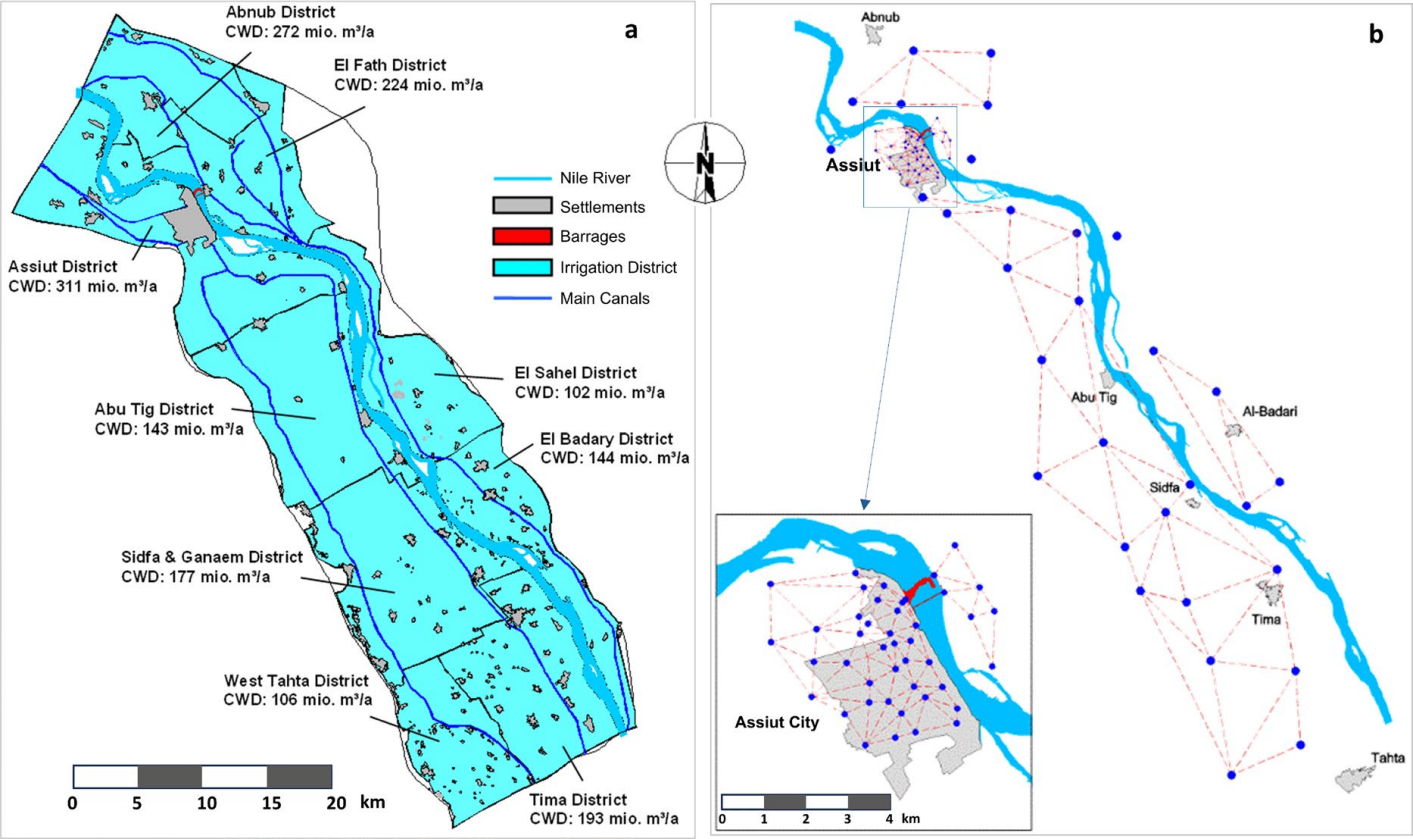
Groundwater study districts upstream and downstream of the project (**a**), and a network of 55 observation wells, bi-monthly readings (**b**)

The present study was conducted during the establishment of the new Assiut Barrages Project in Assiut, Upper Egypt. As part of the Nile Valley, the Assiut area is distinguished by a low-lying region bounded on the east and west by prominent calcareous plateaus, with younger and older alluvial plains forming the morphological units (Said, 1981). It is classified as a semi-arid climate, with exceptionally low annual precipitation, moderate winter temperatures, and extremely high summer temperatures (El-Anwar et al., 2025; Ismail et al., 2023). The study area covers 1113 km^2^, and the new barrage is located ~ 400 m downstream of the existing old barrage. The new barrage is approximately enclosed between latitudes (26° 50′ 00′′ and 27° 20′ 00″ N) and longitudes (30° 40′ 00′′ and 31° 30′ 00″ E), as shown in Fig. 1. A total of 87 wells were drilled in the study area to monitor groundwater levels and to assess the effect of water retained in front of the barrages on groundwater levels (Fig. 1). Fifty-five observation wells across eight districts around the project, both upstream and downstream of the Nile River, were selected. Groundwater levels were monitored regularly every 15 days from 2004 to 2008 and again from 2012 to 2018, using observation wells spread along 65 km upstream and 20 km downstream. We focused on the seventh month (July) to monitor water-level changes in the observed wells over 12 years, as this month marks the summer season—the period of greatest water need.

### Sampling collection and analysis

**Fig. 2:**
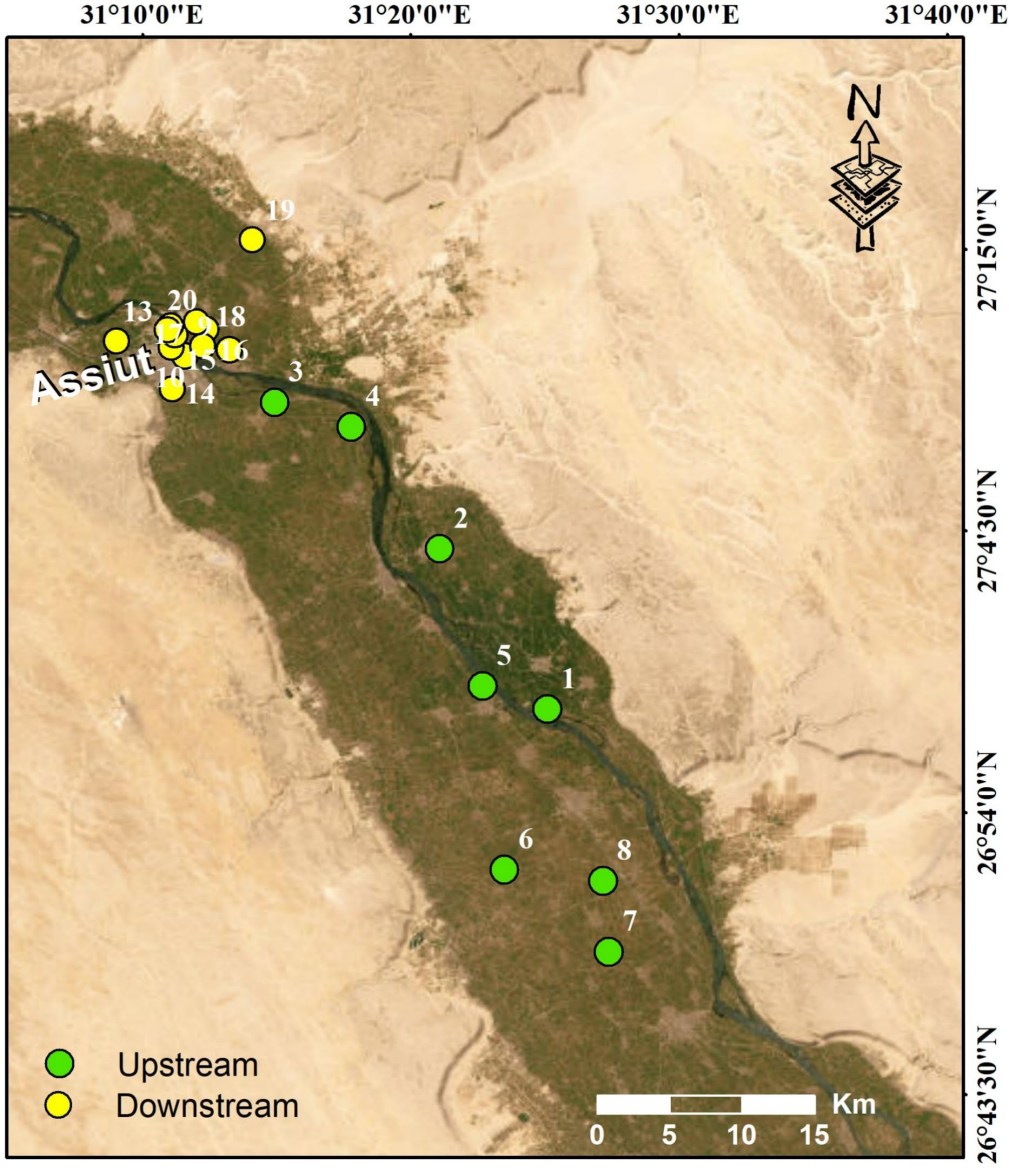
Location map of the sampling sites

In addition to monitoring groundwater levels before and during construction of the new barrage, 20 water samples were collected during 2018 from 20 observation wells to assess groundwater quality in project-affected areas, and to use the observation wells for agricultural purposes after the project’s completion (Figs. 2 and S1; Table S1). The sample collection and analysis were conducted in accordance with standard procedures documented by APHA (2017). The water samples were collected in polypropylene bottles (500 ml). Polypropylene bottles were sealed with Parafilm® and then placed in separate plastic bags to avoid cross-contamination. Water samples were immediately transported to the laboratory in ice boxes and stored at 4 °C for analysis. The collected water samples were chemically analyzed for standard components, major and minor anions and cations. The measured parameters include pH, water temperature, electrical conductivity (EC), dissolved oxygen (DO), ammonium (NH_4_), nitrate (NO_3_), biochemical oxygen demand as O_2_ (BOD), and eight heavy metals e.g., iron (Fe), manganese (Mn), lead (Pb), cadmium (Cd), nickel (Ni), copper (Cu), zinc (Zn), chromium (Cr), arsenic (As) and cobalt (Co).

The various parameters were determined using the standard procedures recommended by APHA (2017) (Table S2). Temperature, pH, DO, and EC were measured in situ using portable sensors. A portable water-quality meter (HORIBA, Ltd., U-10) was used to measure pH, TDS, and DO, while an HQ40D (Hach, USA) was used to determine conductivity. Further, the other parameters were tested in the laboratory, including BOD concentration, determined by measuring decreases in oxygen concentration after a 5-day incubation in the dark at 20 °C. Ammonia (NH_4_^+^-N) was detected by the phenate method, and Nitrate (NO_3_¯-N) was estimated by a UV spectrophotometer (EMCLAB, EMC-11-UV). The heavy metals in the water samples were quantified by the Atomic Absorption Spectrophotometer (AAS; SHIMADZU AA-6800) with a graphite furnace after acid digestion.

### Modeling, QA, QC, and statistics

Two models were applied: one to evaluate groundwater quality for several purposes, and the other to assess health risk. The groundwater quality index (GQI) for irrigation, household use, and livestock purposes was determined using the Water Quality Index 2.0 program developed by the Canadian Council of Ministers of the Environment (CCME, 2017). The GQI was computed using the standard limits set by FAO and EPA guidelines (Ayers & Westcot 1985; EPA 2024; USEPA, 2017). GQI is ranked in ascending order as poor, marginal, fair, good, and excellent, with GQI values of < 45, 45- < 65, 65- < 80, 80- < 95, and 95–100, respectively. The details of the GQI calculation are provided in the supplementary information (SI, Text 1).

The USEPA-recommended health risk assessment (HRA) model was employed to evaluate the potential risks of heavy metals to human health (USEPA, 2011, 2026). These metals can reach the human body via three exposure pathways: ingestion, inhalation, and skin contact, posing both carcinogenic and non-carcinogenic health concerns. In evaluating the health risks related to regular groundwater use by adults and children in the area, we focused solely on skin exposure. The calculated dermal HRA (HRA_*d*_) model is as follows:

1 $$ PDD = \frac{C \times SA \times ABS \times CF \times ET \times KP \times EV \times ED \times EF}{{BW \times AT}} $$

2 $$ HQ = PDD/RfD_{d} $$

3 $$ HI = \sum HQ = \sum PDD/RfD $$

4 $$ CR = PDD \times CSF_{d} $$

5 $$ CR_{T} = \sum CR = \sum PDD \times CSF $$

where PDD, HQ, and CR stand for the probable daily dose of exposure, hazard quotient, and carcinogenic risk of selected metals through dermal contact, respectively. RfD_d_ is the toxicity reference dose, and CSF_d_ is the cancer slope factor through dermal contact. HQ and CR represent the non-carcinogenic and carcinogenic risks from dermal contact with a single metal and were calculated using Eqs. (2) and (4), respectively. The hazard index (HI) and total carcinogenic risk (CR_T_) were used to evaluate non-carcinogenic and carcinogenic risks from all metals (Shomar & Rashkeev 2021; Wang et al., 2025) and were determined using Eqs. (3) and (5), respectively. Details of the exposure parameters and their values are provided in SI (Text 2). The selected parameter values were taken from prior studies (Abdelhafiz et al., 2021a, Shomar & Rashkeev 2021) and are available in the USEPA database (USEPA, 2019).

For quality control and assurance, all reagents and chemicals used were purchased from BDH, Sigma, Aldrich, Merck, Fluka, and Kanto Chemical Co. (Japan) (A.R. 99.9%). The atomic absorption spectroscopic standard solutions (1000 ± 0.002 g/L) for the elements were purchased from Panreac Quimica SA, a company based in the European Union, which is traceable to SRM from NIST. Working standard solutions were prepared by diluting the stock solution using deionized water. For heavy metals, calibration curves were constructed for each element by analyzing standard solutions at appropriate concentrations. The absorbance of the blank was measured prior to sample examination. The statistical analyses were conducted using the R software (version 4.2.1), and the data were presented using the QGIS (version 4.0) and Origin programs.

## Results and discussion

### Behavior of observed groundwater levels

**Fig. 3:**
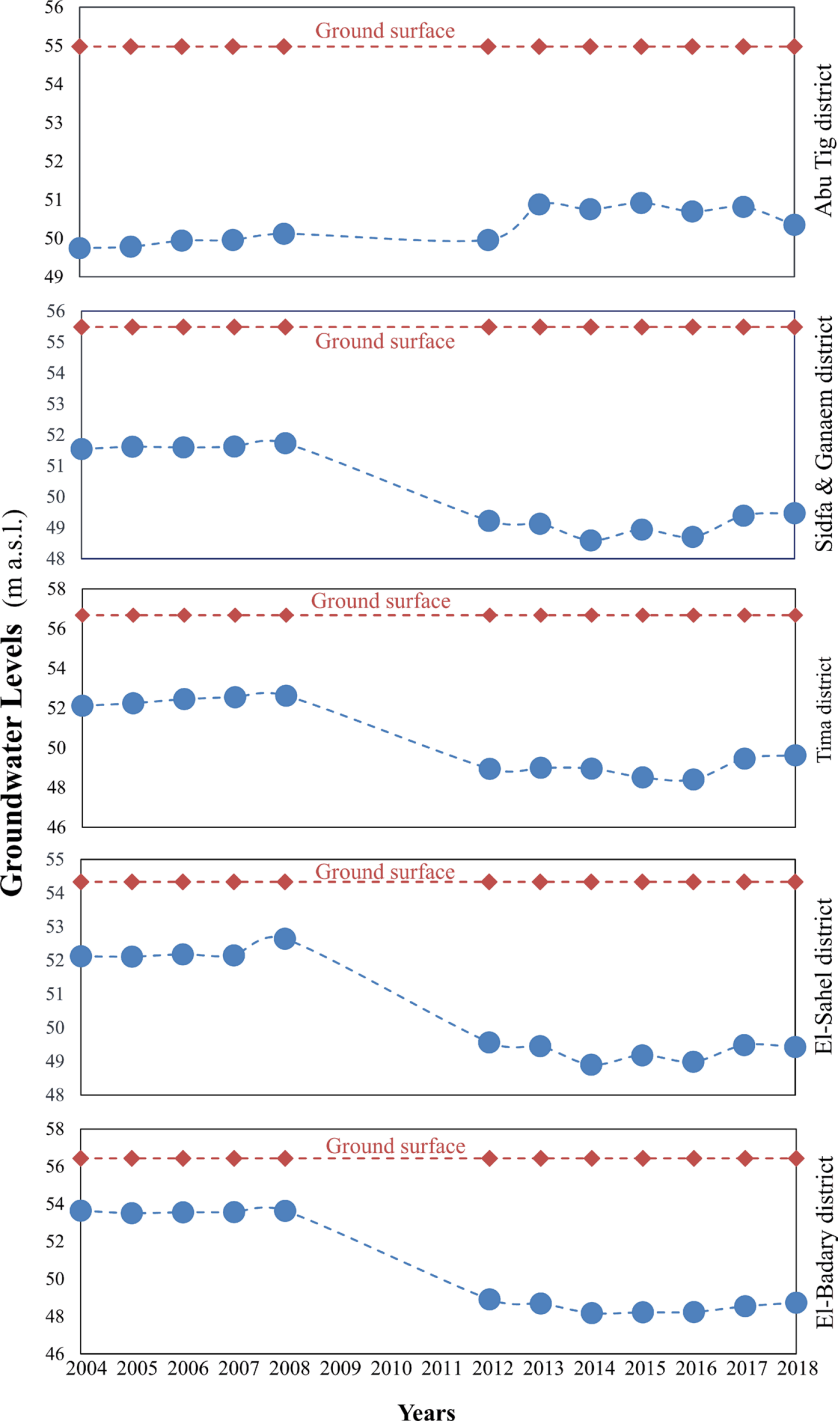
Monitoring groundwater levels in the upstream districts, as explained on the lift axes. m: meter, a.s.l: above sea level

**Fig. 4:**
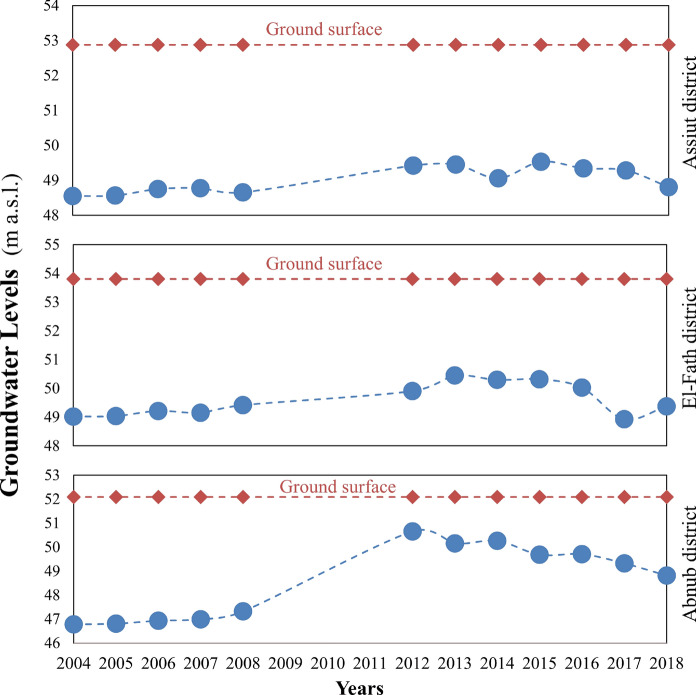
Monitoring the groundwater levels in the downstream districts, as displayed on the lift axes. m: meter, a.s.l: above sea level

**Fig. 5:**
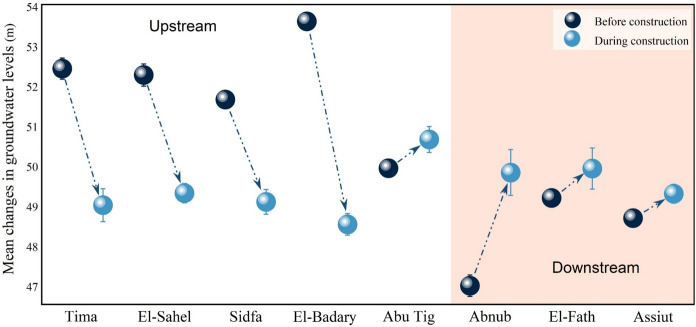
The average groundwater levels in the studied districts before and during the construction of a new barrage. The white background reflects the upstream, and the colored one signifies the downstream

The construction and operation of barrages significantly impact river ecosystems through mechanisms such as water storage, runoff regulation, and control of groundwater recharge to maintain aquifer levels (Kumar et al., 2023; Oh et al., 2016; Zang et al., 2024). Along with the impacts of dams on reservoir groundwater levels and on the sustainability of groundwater and agriculture (Ashraf et al., 2007; Çelik, 2018; Sulistiyono et al., 2021), the direct impact of dams on groundwater is expected to occur across upstream and downstream areas. Therefore, it is essential to study their impact on groundwater levels and quality. The behavior of groundwater due to the new barrage could be discussed in eight districts. Five of which represent the upstream areas on the east and west banks of the Nile River, include Tima district, Sidfa & Ganaem district, El-Sahel district, El-Badary district, and Abu Tig district, and are distributed along 65 km around the project. The other three districts lie downstream and encompass Assuit, El-Fath, and Abnub. Groundwater levels in both upstream and downstream districts were monitored and illustrated in Figs. 3–5. The average changes in groundwater levels are shown in Fig. 5, with dashed lines and arrows indicating whether levels were rising or falling. Firstly, the behavior of observed groundwater levels upstream of the Nile River, before and during the construction of the new barrage, is discussed below and shown in Fig. 3.

Groundwater levels in the Tima district before new barrage construction were 52.12, 52.25, 52.45, 52.55, and 52.62 m a.s.l. in 2004, 2005, 2006, 2007, and 2008, respectively. During the construction period, the water levels were 48.94, 48.99, 48.95, 48.51, 48.41, 49.46, and 49.62 m a.s.l. in 2012, 2013, 2014, 2015, 2016, 2017, and 2018, respectively (Fig. 3). Groundwater levels at the Tima district during the construction of a new barrage decreased by about 3.41 ± 0.23 m compared to before construction (Fig. 5). Similar patterns were observed in the Sidfa & Ganaem, El-Sahel, and El-Badary districts. The average groundwater levels in the Sidfa & Ganaem district from 2004 to 2008 and from 2012 to 2018 were 51.62 ± 0.23 and 49.07 ± 0.33 m a.s.l., respectively. The ground surface elevation in that district was 55.48 m above sea level (Fig. 3). Furthermore, the mean levels of groundwater before and during the construction of the new barrage in El-Sahel district were 52.24 ± 0.23 and 49.28 ± 0.26 m a.s.l., respectively. While the average groundwater elevation in El-Badary district was 53.58 ± 0.06 m above sea level from 2004 to 2008, it was 48.50 ± 0.06 m above sea level from 2012 to 2018. The elevations of the ground surface in El-Sahel and El-Badary districts were 54.33 and 56.43 m above sea level, respectively (Fig. 3). During the construction of a new barrage, groundwater levels in Sidfa & Ganaem, El-Sahel, and El-Badary districts fell by approximately 2.56 ± 0.27, 2.95 ± 0.04, and 5.08 ± 0.24 m relative to pre-construction levels (Fig. 5).

The average difference in groundwater levels before and during construction in the Abu Tig district was 0.72 ± 0.2 m. In addition to the new barrage construction, slightly higher groundwater levels in the Abu Tig district compared to other upstream districts might also be due to its proximity to Assiut city, as the water is held back behind the new barrages upstream. Dams increase upstream groundwater levels by impounding water, which exerts direct hydraulic pressure on the water table and by infiltrating into aquifers, raising the water table in the nearby district (Sulistiyono et al., 2021). Çelik (2018) studied the effects of the Kralkızı and Dicle dams in Turkey on groundwater from 1996 to 2011 and found significant impacts on rising water levels, especially on the near-upstream and downstream sides, with effects decreasing with distance from the dams.

Generally, we observed declining water levels in the observation wells across all study districts in the upstream area, except in the Abu Tig district. The lowest elevation during the new barrage construction was recorded in the El-Badary district, with the trend in groundwater levels at the present sites in the upstream area in the order of El-Badary district < Tima district < El-Sahel district < Sidfa & Ganaem district < Abu Tig district. This might be due to excessive water withdrawals from wells dug by farmers for irrigation, leading to a drawdown of the surface water layer. It has been cited that intensive groundwater pumping for agricultural irrigation led to significant declines in groundwater levels and streamflow over time (Barlow & Clark 2011, Barlow & Leake 2012, Killian et al., 2019). In addition, the water supply to the surface layer of these wells in upstream areas is scarce because surface waters in these districts are at the ends of canals, especially in the Tima district.

Regarding groundwater levels in the observation wells across the other districts in the downstream area, they were recorded at 48.55, 48.56, 48.75, 48.77, and 48.66 m above sea level before construction in 2004, 2005, 2006, 2007, and 2008, respectively, in the Assiut district. In 2012, 2013, 2014, 2015, 2016, 2017, and 2018, during construction, it was 49.42, 49.45, 49.06, 49.53, 49.34, 49.28, and 48.81 m a.s.l., respectively, with a ground surface elevation of 52.88 m a.s.l. (Fig. 4). The average groundwater levels prior to and during the construction of the new barrage in the Abnub district were 46.97 ± 0.22 m a.s.l. and 49.80 ± 0.62 m a.s.l., respectively. The average groundwater elevation in the El-Fath district was 49.17 ± 0.16 m above sea level from 2004 to 2008, and 49.90 ± 0.56 m above sea level from 2012 to 2018. The ground surface elevations in the Abnub and El-Fath districts were 52.09 m and 53.80 m above sea level, respectively (Fig. 4). Groundwater levels in Assiut, Abnub, and El-Fath districts increased by roughly 0.61 ± 0.15 m, 2.83 ± 0.40 m, and 0.73 ± 0.40 m, respectively, compared to pre-construction levels during the barrage construction (Fig. 5).

Measured groundwater levels were used to determine groundwater behavior in each district around the project, spanning 20 km downstream of the Nile River in three districts: Assiut, El-Fath, and Abnub. We noted that water levels in observation wells increased downstream in all study districts during the construction of the new barrage, with the highest elevation recorded in the Abnub district, followed by El-Fath and Assiut (Fig. 5). This may be due to a decrease in groundwater extraction caused by a reduction in cultivated areas in downstream districts, which are mainly urban rather than extensive agricultural sectors that require irrigation and water use, especially in Assuit and El-Fath districts. Additionally, impacts on downstream groundwater-level fluctuations are reflected in temporal changes in groundwater levels measured in observation wells. Prior studies show that groundwater-storage dams temporarily release water into the environment and recharge the subsurface reservoirs, leading to larger fluctuations in downstream groundwater levels (Liu et al., 2023; Mori et al., 1997).

Previous studies highlight the impacts of barrages on elevating groundwater tables, leading to agricultural and urban waterlogging (Dawoud & Allam 2004; Dawoud et al., 2006; Yihdego, 2017). Barrages increased groundwater levels beneath urban structures, adversely affecting buildings and sanitation facilities, and in agricultural areas, they caused secondary salinization of the soil profile. They highlighted the importance of understanding the barrage’s effects, offered mitigation measures, and presented an appropriate approach to overcoming them (El-Fakharany & Fekry 2014; Yihdego, 2016; Yihdego et al., 2017). In addition to the impacts of barrages, other hydro-agricultural stressors, such as irrigation abstraction and land‑use change, were previously highlighted. It is estimated that worldwide groundwater reserves are being rapidly depleted to meet irrigation demands for agricultural output (Konikow, 2015; Siebert et al., 2010). Excessive withdrawals have caused substantial declines in groundwater levels, threatening the aquifer’s long-term water supply and raising serious concerns about the sustainability of groundwater resources (Feng et al., 2024; Lovelace et al., 2020). Another stressor is land-use change, which affects the entire hydrological system by altering surface water runoff, infiltration, and groundwater recharge (Feng et al., 2024; Jinno et al., 2009). Salem et al. (2023) emphasized the decline in groundwater recharge caused by land-use changes, which reduced recharge to both shallow and deep aquifers and heightened environmental risks.

Overall, constructing barrages significantly affects groundwater levels, especially around them on the upstream and downstream sides and near the reservoir. The water level in the barrages’ basins rises dramatically, then stabilizes at a fixed level, while the dams’ effects on basins far from them diminish. Our study identified the influence of barrage construction, which may increase groundwater levels in Assiut city and in some upstream areas, requiring proper management to mitigate environmental impacts.

### Groundwater quality assessment

Groundwater quality is a crucial environmental issue worldwide and is primarily influenced by physicochemical parameters (Abdelhafiz et al., 2021b; Tiwari et al., 2018). A thorough assessment of groundwater’s physicochemical properties, quality, and associated health risks provides reliable evidence for its use (Li et al., 2023). Therefore, groundwater quality characterization should be conducted before decisions are made on water allocation for different uses. The following sections identify the physicochemical and heavy metal parameters for twenty observations, both upstream and downstream of the new Assuit barrage on the River Nile. Groundwater quality for different purposes, particularly irrigation, and associated health risks were also evaluated.

#### Physicochemical and heavy metal parameters

The chemical compositions of the water samples from observation wells, including physical, chemical, and heavy metals, are given in Table 1. The measured pH from upstream observation wells ranged from 7.1 to 8.9, with an average of 8.2, and from 7.3 to 8.3 downstream, with an average of 8. This indicates groundwater is nearly neutral to alkaline, with most samples mainly alkaline. The average (range) EC levels were 1328 (660–1990) and 1026 (539–1498) µS/cm in the upstream and downstream, respectively (Table 1), indicating multiple processes occurring in the groundwater system (Rao, 2016). Similar to EC, higher TDS values were recorded in upstream wells, averaging 907 mg/L, compared with 781 mg/L in downstream wells. This might be due to extensive agricultural practices in the upstream area, with agricultural leakage, which elevates water salinity (Foster, 2022; Salman et al., 2019; Suarez, 1989). NO_3_^−^ oncentrations, which mainly originate from agricultural compost and animal waste (He et al., 2019; Rahmati et al., 2015), in groundwater samples from the studied area ranged from 1.89 to 10.58 mg/L, with an average of 4.47 mg/L in upstream samples and 2.23 to 20.7 mg/L, with an average of 6.5, in downstream samples (Table 1). NH_4_^+^ levels were significantly higher in groundwater from the downstream compared to the upstream. The average NH_4_^+^ in groundwater was 1.21 mg/L downstream and 0.3 mg/L upstream. The higher NH_4_^+^ levels downstream than upstream might result from domestic wastewater seepage, given the area's urban environment (Abdelhafiz et al., 2021b; Norrman et al., 2015). High F levels in groundwater (0.6–1.5 mg/L) are a major issue in many regions worldwide, putting about 200 million people at risk (Ayoob & Gupta 2006). Comparable F levels were observed in both upstream and downstream groundwater. The mean F contents were 0.51 mg/L in the upstream and 0.52 mg/L in the downstream, meeting safe F limits (Table 1).

**Table 1:** Physical, chemical, and heavy metals parameters of groundwater for twenty observation wells of the new Assuit Barrage, the units of Fe, Mn, Cu, and Zn in mg/L, while Cd, Ni, Cr, and Al in µg/L

Sample No		pH	EC	TDS	DO	COD	TOC	NO_3_	NH_4_	F	Fe	Mn	Zn	Cu	Al	Cd	Ni	Cr
1	Upstream	7.1	965	648	1.68	51.2	4.9	1.89	0.53	0.59	2.15	0.27	0.89	0.11	72	0.02	8.22	2.29
2		8.2	1932	1347	3.84	22.4	4	4.77	0.02	0.6	2	0.47	0.17	0.07	101	0.01	8	3.08
3		8.4	744	491	0.88	19.2	4.5	4.07	0.47	0.34	20.63	0.79	0.14	0.07	183	0.03	2.09	4.44
4		8.9	1617	1099	1.05	44.8	11.3	4.96	0.84	0.56	1.8	0.11	0.11	0.04	146	0.01	2.58	4.55
5		8.1	1691	1157	0.21	35.2	9.6	3.09	0.12	0.36	13.02	0.82	0.10	0.13	136	0.03	4.56	4.43
6		8.1	660	436	3.12	22.4	4.4	2.52	0.15	0.52	5	1.83	0.35	0.21	147	0.13	2.28	1.52
7		8.4	1021	688	2.01	32	6	3.85	0.15	0.56	2.14	0.53	0.14	0.10	142	0.06	3.86	1.52
8		8.4	1990	1392	1.33	51.2	10.1	10.58	0.20	0.57	5.2	1.20	0.36	0.16	147	0.21	2.89	4.29
	Mean	8.2	1328	907	1.77	34.8	6.85	4.47	0.31	0.51	6.49	0.75	0.28	0.11	134	0.06	4.31	3.27
9	Downstream	8.3	893	596	2.12	32	6.9	2.66	1.49	0.77	2.86	0.25	0.08	0.07	144	0.14	3.21	3.74
10		8.2	757	495	3.27	32	5.3	2.06	0.529	0.62	1.41	0.22	0.18	0.06	146	0.16	1.68	4.97
11		8.3	539	352	0.29	44.8	7.6	2.88	0.544	0.58	5.18	0.76	0.06	0.08	146	0.13	1.34	3.78
12		8.0	1498	1025	1.24	41.6	11.0	16.19	4.238	0.64	2.59	0.83	0.23	0.10	184	0.16	3.21	4.03
13		7.7	1225	829	0.85	112	123.0	4.44	4.158	0.43	3.8	0.62	0.30	0.11	171	0.27	1.91	2.39
14		8.3	1077	727	1.85	64	98.0	2.41	0.306	0.59	1.12	0.16	0.24	0.06	128	0.08	2.29	2.36
15		8.3	1171	790	0.0	60.8	129.0	20.7	0.681	0.48	29.31	9.65	3.50	0.23	76	1.66	1.98	0.80
16		7.3	1169	1882	0.79	9.6	123.0	5.73	0.03	0.31	2.61	0.78	0.06	0.06	183	0.13	30.08	10.28
17		8.3	1173	792	1.17	32	122.0	2.23	0.03	0.51	1.36	0.71	0.29	0.08	143	0.08	1.33	0.87
18		7.8	855	569	0.25	6.4	133.0	9.68	0.001	0.38	18.17	1.49	0.19	0.11	144	0.18	1.06	1.36
19		7.9	950	635	0.43	192	164.0	2.82	1.112	0.32	7.1	1.79	0.28	0.08	183	0.27	4.40	2.44
20		7.9	1010	678	0.13	67.2	110.0	6.23	1.391	0.6	9.12	0.66	0.13	0.11	183	0.41	2.92	3.33
	Mean	8.0	1026	781	1.03	57.9	86.1	6.5	1.21	0.52	7.05	1.49	0.46	0.10	153	0.30	4.62	3.36
Limits*	Irrigation	8.5	2000	1400	–	–	–	20	5	1	5	0.2	2	0.2	5000	10	200	100
	Household	8.5	1500	1000	–	–	–	50	0.5	2	0.3	0.05	5	1.3	200	3	70	50
	Livestock	8.5	5000	3000	–	–	–	100	10	2	0.3	0.1	24	0.5	5000	10	1000	1000

^*^The maximum limits according to the Food and Agriculture Organization (FAO) and the U.S. Environmental Protection Agency (EPA)

The concentrations of heavy metals, including Al, Fe, Mn, Zn, Cu, Cd, Ni, and Cr in groundwater are listed in Table 1. Fe concentrations, the second most abundant metallic element in the Earth’s crust and probably transported by erosion, leaching, and the movement of solid particles, ranged from 1.8 to 20.63 mg/L, with an average of 6.49 mg/L in upstream and between 1.12 and 29.31 mg/L, and the average was 7.05 mg/L in downstream. Mn is the fifth most abundant metal on Earth and primarily results from the leaching of Mn from rocks and soils (WHO, 2017). Mean Mn concentration was higher downstream than upstream, ranging from 0.11 to 1.83 mg/L, with an average of 0.075 mg/L upstream and between 0.16 and 9.65 mg/L downstream, averaging 1.49 mg/L (Table 1). The Cu measured in the groundwater samples ranged from 0.04 to 0.21 mg/L in upstream samples and from 0.07 to 0.23 mg/L in downstream samples, with average values of 0.11 mg/L and 0.10 mg/L, respectively. It reflects the analogous Cu content of upstream and downstream groundwater. Zn levels in groundwater samples from the studied area ranged from 0.10 to 0.89 mg/L, with an average of 0.28 mg/L upstream, and between 0.06 and 3.5 mg/L, with an average of 0.46 mg/L downstream.

Cd is a highly toxic trace element with a long half-life and is present relatively rarely in the Earth, with the average concentration being 0.2 μg/g in the Earth’s crust (McGeer et al., 2011). Cd ranged from 0.01 to 0.21 µg/L, with an average of 0.06 µg/L in upstream samples and between 0.08 and 1.66 µg/L, with an average of 0.30 µg/L in downstream samples (Table 1). Cr is present in small quantities in nature and is usually found at very trace concentrations in groundwater, not influenced by point-source contamination (Zhou et al., 2019). Cr concentrations in groundwater samples of the studied area ranged from 1.52 to 4.55 µg/L, with an average of 3.27 µg/L in upstream and between 0.80 and 10.28 µg/L, with an average of 3.36 µg/L in downstream. Ni concentrations ranged from 2.09 to 8.22 µg/L with an average of 04.31 µg/L in upstream and between 1.06 and 30.8 µg/L, with an average of 4.62 µg/L in downstream. Al is the most widely distributed metal in the environment and is extensively used in modern daily life. Acid rain has caused elevated Al levels in many freshwater sources (Lawrence et al., 2007). Al varied from 72 to 183 µg/L upstream, with an average of 134 µg/L, and from 76 to 184 µg/L downstream, with an average of 153 µg/L (Table 1).

**Fig. 6:**
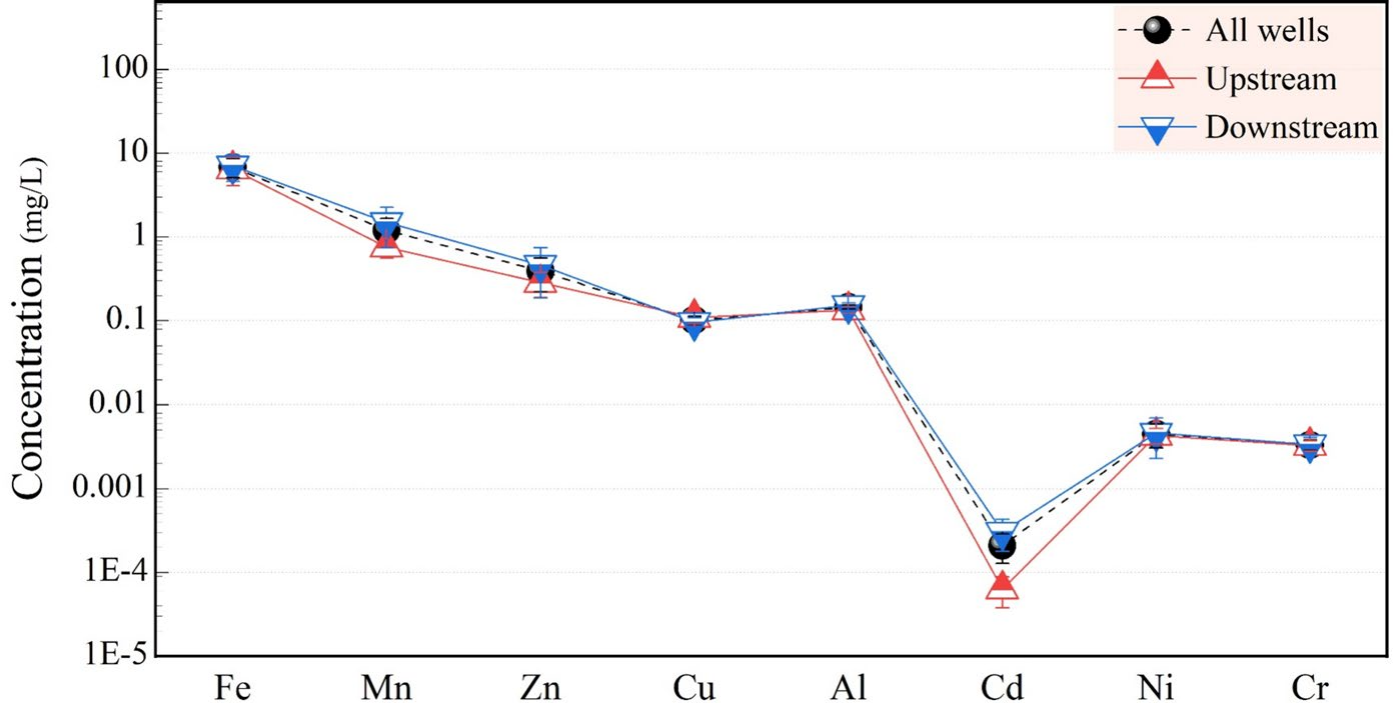
Comparison of heavy metal concentrations in groundwater from overall, upstream, and downstream wells. Each plot bar represents the standard error (± SE)

Mean heavy metal concentrations in groundwater samples from all studied wells are in the sequence of Fe > Mn > Zn > Al > Cu > Ni > Cr > Cd (Fig. 6). Similar orders were also observed in both the upstream and downstream, with the highest metal being Fe (6.49 ± 2.4 at upstream and 7.05 ± 2.45 at downstream) and the lowest being Cd (0.00006 ± 0.00003 at upstream and 0.0003 ± 0.0001 at downstream). Dashed lines in Fig. 6 connect the average metal concentrations from all wells, while upstream and downstream averages are connected by straight lines. Average heavy metal concentrations were higher in downstream wells than in upstream wells (Fig. 6). It is observed that heavy metal levels across all wells are similar, except for well no. 15 (Table 1). The elevated metal concentrations at that well might be due to its proximity to the sewage treatment plant serving the workers involved in the current barrage project, which has caused downstream groundwater heavy metal levels to exceed those upstream.

#### Quality assessment for different purposes

Quality assessment is crucial for evaluating groundwater for irrigation, given that groundwater in the study region is heavily used for this purpose. The appropriateness of groundwater for irrigation depends on its mineral content and its effects on soil and plants (Richards, 1954; Singh et al., 2009). The study area is under stress due to interactions between rock elements and groundwater, which affect groundwater geochemistry. The physical, chemical, and heavy-metal parameters of groundwater from 20 observation wells located upstream and downstream of the New Assuit Barrage on the River Nile were evaluated for irrigation, household, and livestock purposes.

**Fig. 7:**
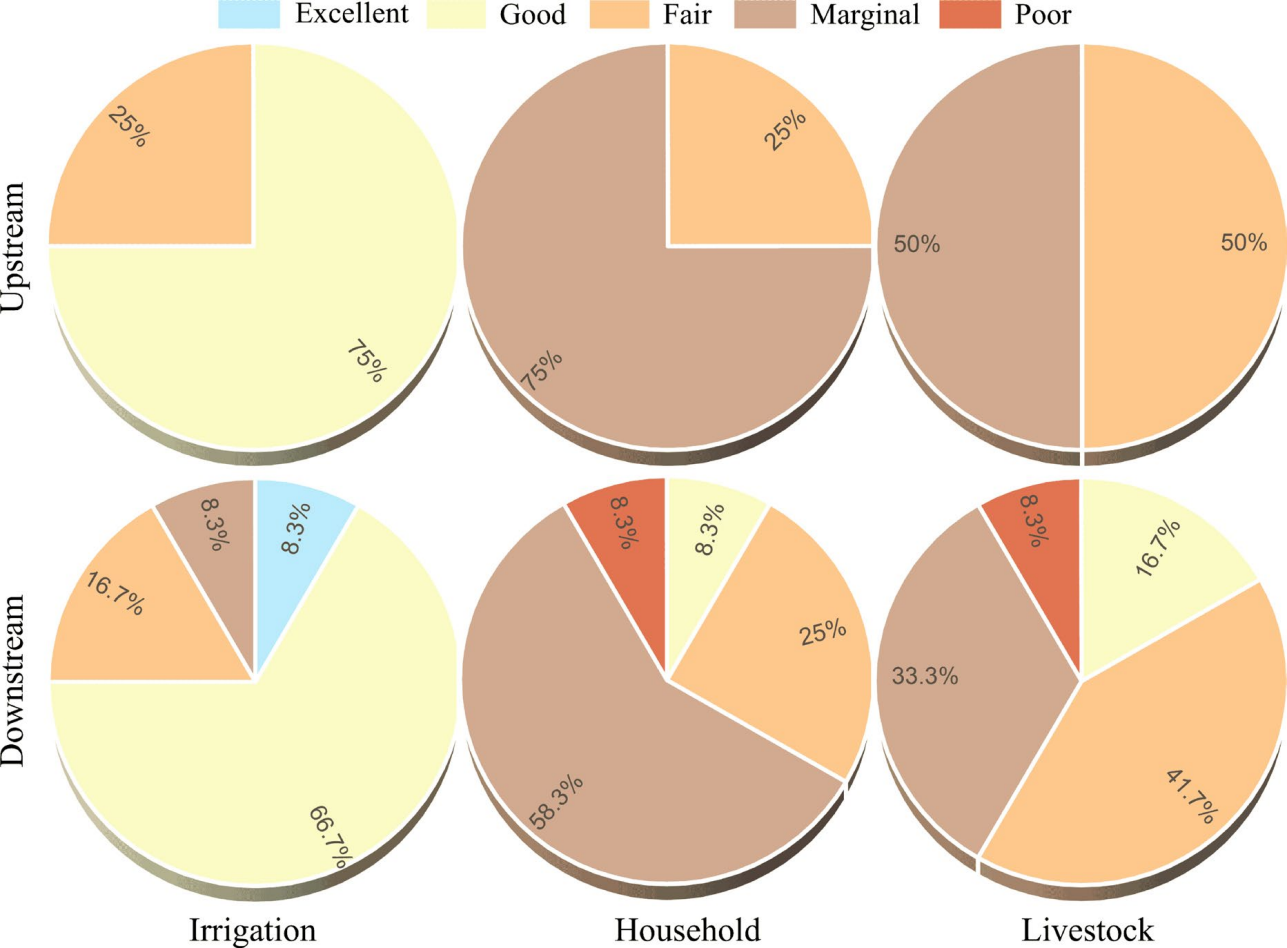
Percentage of groundwater quality index (GQI) categories for irrigation, household, and livestock, in upstream and downstream wells

Groundwater salinity, expressed by EC and TDS, plays a significant role in crop growth. Most wells in the upstream and downstream areas are within permissible limits according to salinity classification, EPA, and FAO standards for irrigation water (Tables S3) (Ayers & Westcot 1985, EPA 2024, Richards, 1954). The relatively high salinity in some wells may be due to the lithological composition of these locations and to the leaching and dissolution of soil salts and chemical fertilizers by irrigation water. The groundwater quality index (GQI) is closely related to human health, as people can be exposed to pollutants in groundwater via drinking and dermal contact (Jadoon et al., 2024). GQI based on measured parameters at each well is shown in Fig. 7 and Table 2. 75% of groundwater samples from upstream wells are in the good category, and the remaining 25% are in the fair category (Fig. 7). The overall water quality index upstream is fair and improves to good quality when Mn is excluded (Table 2), reflecting Mn’s impact on groundwater quality. Although groundwater Mn has a natural, geogenic origin, human activities also contribute to its release into aquifers. Industrialization and urbanization have increased Mn levels in groundwater, mainly from anthropogenic sources such as industrial effluents and sewage discharges (Zhao et al., 2023). Excessive groundwater extraction can elevate Mn levels through inducing anaerobic conditions in aquifers and substantially reducing groundwater levels, thereby facilitating water–rock interactions that release bioavailable Mn. Additionally, optimal reducing conditions in shallow aquifers can lead to elevated Mn concentrations (Hasan & Ali 2010; Shamsuddin et al., 2014). Generally, the findings align with a recent study on water quality in the River Nile near our research area, which reported fair water quality for irrigation (Abdelhafiz et al., 2021a).

**Table 2:** Categorization of groundwater samples for irrigation, household, and livestock in the upstream and the downstream relative to groundwater quality index (GQI) and their overall values

	Well No	Irrigation		Household		Livestock	
		GQI	Category	GQI	Category	GQI	Category
Upstream	1	94	Good	69.7	Fair	76.3	Fair
	2	92.3	Good	62.3	Marginal	74.1	Fair
	3	79	Fair	49.3	Marginal	50	Marginal
	4	94.2	Good	60	Marginal	76.6	Fair
	5	81.4	Good	47.9	Marginal	53.5	Marginal
	6	75.7	Fair	53.2	Marginal	57.8	Marginal
	7	91.6	Good	67.3	Fair	72.7	Fair
	8	80.7	Good	51.3	Marginal	60.1	Marginal
Overall	79	Fair	49.9	Marginal	59	Marginal	
Excluding Mn	86.1	Good	56.7	Marginal	63.1	Marginal	
Downstream	9	94.1	Good	65.9	Fair	73.3	Fair
	10	94.2	Good	73.9	Fair	81.1	Good
	11	84.8	Good	56.7	Marginal	62.3	Marginal
	12	87.9	Good	54	Marginal	68	Fair
	13	90.5	Good	56.8	Marginal	66.3	Fair
	14	100	Excellent	81.1	Good	84	Good
	15	45.8	Marginal	42.2	Poor	44.9	Poor
	16	84.1	Good	60	Marginal	68.4	Fair
	17	89.3	Good	66.4	Fair	73.7	Fair
	18	74.5	Fair	48.8	Marginal	50.1	Marginal
	19	75.5	Fair	50.2	Marginal	55.8	Marginal
	20	84.3	Good	53	Marginal	57.2	Marginal
Overall	67.6	Fair	50	Marginal	56.7	Marginal	
Excluding Mn	77.3	Fair	59.3	Marginal	62.9	Marginal	
Excluding No. 15	82.8	Good	55.2	Marginal	62.7	Marginal	

On the other hand, the calculated GQI for irrigation in the downstream wells indicated that 66.7% of the samples had good quality. The remaining are in the excellent, marginal, and fair categories, with percentages of 8.3%, 8.3%, and 16.7%, respectively (Fig. 7). The overall GQI value downstream is 67.6 (fair), with a 10 percent increase after excluding Mn; however, it still falls within the fair category (Table 2). Well No. 15 had the lowest water quality (marginal) and significantly impacts water quality because it is more affected by wastewater leaching, which raised heavy metal levels. Excluding well 15 from GQI inputs improved the groundwater quality to a good category (Table 2).

Groundwater quality showed similar categories for household and livestock uses both upstream and downstream, with different percentages for each category (Fig. 7 and Table 2). Fair and marginal water quality were present upstream, with 25% categorized as fair and 75% as marginal for households, and 50% categorized as fair and similarly as marginal for livestock. The GQI results for household purposes in the downstream area were 8.3% good, 25% fair, 58.3% marginal, and 8.3% poor quality (Fig. 7). Similarly, for livestock utilization, groundwater quality in the downstream area was categorized into four water levels: good, fair, marginal, and poor, with percentages of 16.7%, 41.7%, 33.3%, and 8.3%, respectively (Fig. 7). The poorest water quality was also recorded in well 15 for both household and livestock practices (Table 2). The overall GQI in both upstream and downstream areas was marginal for household and livestock use, indicating the need for effective treatment before using groundwater for these purposes (Table 2).

#### Dermal health risk assessment

**Fig. 8:**
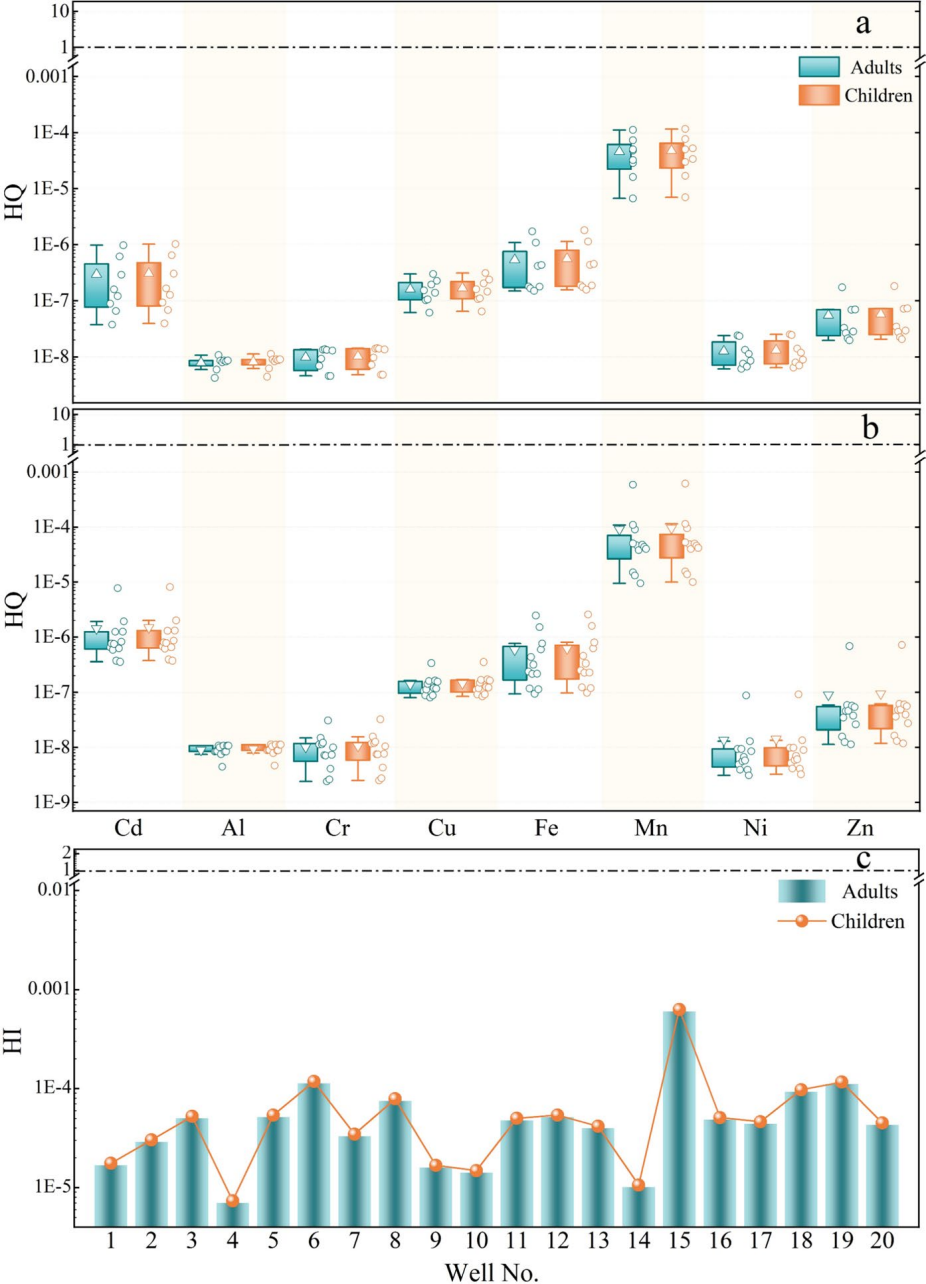
Hazard quotient (HQ) for dermal exposure to toxic elements in upstream (**a**) and downstream (**b**) groundwater, along with the hazard index (HI) for all samples (**c**), were plotted on a log scale for adults and children

Clean groundwater, especially in arid and semi-arid regions, is essential for food safety and public health (Gao et al., 2022). Excessive groundwater use with elevated levels of toxic elements may lead to their accumulation in the human body, either through ingestion or skin absorption, ultimately entering the bloodstream and posing serious health risks. Most exposure to groundwater occurs through skin contact; therefore, a thorough assessment of heavy metal hazards in groundwater via dermal exposure to human health is urgently needed to guide groundwater quality protection and risk management. Carcinogenic and non-carcinogenic risk results from dermal exposure of adults and children to heavy metals in upstream and downstream groundwater are presented in Fig. 8 and Table 3. Additionally, the detailed results for non-carcinogenic risk (HQ and total HQ as HI) and carcinogenic risk (CR and total CR_T_) for each sample are provided in tables S4–S7. HQ results were shown in Fig. 8 as colorless circles to the left of each box, and the average HQ results are plotted as a colorless triangle. Dashed-dotted lines indicate 1 for HQ and HI, as consumers exposed to HQ/HI values below 1 are deemed safe. If HQ and HI values are 1 or higher, they are considered unsafe for human health and indicate a potential health risk; thus, appropriate protective measures should be taken.

**Table 3:** Results of health risks from dermal contact with groundwater in upstream and downstream areas for adults and children; data shown as mean (range)

	Upstream		Downstream					
	HQ	CR	HQ	CR				
*Adults*								
Cd	2.96E-07 (3.8E-08- 9.9E-07)	3.94E-11 (5E-12-1.3E-10)	1.43E-06 (3.6E-07-7.8E-06)	1.90E-10 (4.7E-11-1.0E-09)				
Al	7.88E-09 (4.2E-09-1.1E-08)		8.95E-09 (4.5E-09-1.1E-08)					
Cr	9.82E-09 (4.6E-09-1.4E-08)	4.23E-08 (2E-08-5.9E-08)	1.01E-08 (2.4E-09-3.1E-08)	4.36E-08 (1.0E-08-1.3E-07)				
Cu	1.61E-07 (6.2E-08-3.0E-07)		1.4E-07 (8.1E-08-3.4E-07)					
Fe	5.44E-07 (1.5E-07-1.7E-06)		5.91E-07 (9.4E-08-2.5E-06)					
Mn	4.59E-05 (6.7E-06-1.1E-04)		9.12E-05 (9.5E-06-5.9E-04)					
Ni	1.26E-08 (6.1E-09-2.4E-08)	1.61E-10 (7.8E-11-3.1E-10)	1.35E-08 (3.1E-09-8.8E-08)	1.73E-10 (4E-11-1.1E-09)				
Zn	5.53E-08 (2.0E-08-1.7E-07)		9.04E-08 (1.1E-08-6.8E-07)					
∑total	HI	4.7E-05 (7E-06-1E-04)	CR_T_	4.25E-08 (2E-08-6E-08)	HI	9.35E-05 (1E-05-6E-04)	CR_T_	4.39E-08 (1E-08-1E-07)
*Children*								
Cd	3.1E-07 (3.9E-08-1.0E-06)	4.21E-12 (5.3E-13-1.4E-11)	1.50E-06 (3.7E-07-8.1E-06)	2.03E-11 (5.1E-12-1.1E-10)				
Al	8.25E-09 (4.4E-09-1.1E-08)		9.37E-09 (4.7E-09-1.1E-08)					
Cr	1.03E-08 (4.8E-09-1.4E-08)	4.52E-09 (2.1E-09-6.3E-09)	1.06E-08 (2.5E-09-3.2E-08)	4.65E-09 (1.1E-09-1.4E-08)				
Cu	1.68E-07 (6.5E-08-3.1E-07)		1.46E-07 (8.4E-08-3.5E-07)					
Fe	5.7E-07 (1.6E-07-1.8E-06)		6.19E-07 (9.8E-08-2.6E-06)					
Mn	4.81E-05 (7.0E-06-1.2E-04)		9.55E-05 (1.0E-05-6.2E-04)					
Ni	1.32E-08 (6.4E-09-2.5E-08)	1.72E-11(8.3E-12-3.3E-11)	1.42E-08 (3.3E-09-9.2E-08)	1.84E-11 (4.2E-12-1.2E-10)				
Zn	5.79E-08 (2.1E-08-1.8E-07)		9.47E-08 (1.2E-08-7.2E-07)					
∑total	HI	4.93E-05(7E-06-1E-04)	CR_T_	4.54E-09(2E-09-6E-09)	HI	9.79E-05(1E-05-6E-04)	CR_T_	4.69E-09(1E-09-1E-08)

HQ = Hazard quotient, HI = Hazard index, CR = Carcinogenic risk, CR_T_ = Total CR

The calculated HQ is quite small for all metals in both streams for adults and children, and was well below 1 (Fig. 8a and b). The slightly elevated HQ in children may be attributed to the organ development, diminished metabolic and detoxification abilities, and several behavioral factors (Sun et al., 2023; Wang et al., 2025). Although HQ values for Mn remained within safe limits, they were the highest among the metals tested in both the upstream and downstream areas (Table 3). Extreme exposure to Mn poses a serious health risk (Ramachandran et al., 2021). Overexposure to Mn can affect brain health, lead to intellectual impairment (Bouchard et al., 2010; Miah et al., 2020), and cause Alzheimer’s disease, mitochondrial dysfunction, cardiovascular disease, sperm damage, and other respiratory and neurological disorders (Menezes-Filho et al., 2009; Rushdi et al., 2023; Wasserman et al., 2005; Williams et al., 2013). This exposure can be severe and harmful, and although children are considered especially vulnerable, Mn exposure is not confined to any single age group or gender (Miah et al., 2020). The quality and health risk results underscore the need for further investigation to clarify Mn patterns in groundwater, identify potential risks, and mitigate Mn more effectively.

Similarly, the highest HI for groundwater was observed in the 15th well (Fig. 8c), underscoring the need to monitor groundwater quality to mitigate potential risks. Moreover, Table 3 shows that the calculated carcinogenic and total carcinogenic values are below 1E-6, indicating that the dermal exposure risks from heavy metals in all groundwater wells are negligible. Conversely, previous studies have identified both non-carcinogenic and carcinogenic risks associated with oral intake of groundwater contaminated by heavy metals (Abdelhalim et al., 2023; Salem et al., 2025). This variation arises from differences in location, pollution sources, and exposure routes. Overall, our findings show that both carcinogenic and non-carcinogenic risks from dermal contact are minimal.

## Conclusion and implication

The specific impact of the new barrage on groundwater behavior was assessed using extensive long‑term monitoring of groundwater levels from 55 observation wells across eight districts. To sustainably manage groundwater resources, control contamination, and mitigate its effects on soil, plants, and human health, the groundwater quality index (GQI) and human health risks (HRA) were evaluated. Constructing barrages significantly affected groundwater levels, especially around the upstream and downstream barrages and near the reservoir. Groundwater levels in the upstream districts generally decreased, except in the Abu Tig district, which is near the constructed barrage that retains water. Excessive abstraction of groundwater for irrigation, combined with a scarcity of water in the surface layer of these wells, as the upstream districts are the ends of canals, led to declining groundwater levels. In contrast, during construction of the new barrage, groundwater levels in downstream districts, including Assiut, Abnub, and El-Fath, rose by roughly 0.61 ± 0.15 m, 2.83 ± 0.40 m, and 0.73 ± 0.40 m, respectively, relative to pre-construction levels. Lowering groundwater extraction due to reduced cultivated areas in downstream districts, which are mainly urban rather than agricultural, led to rising groundwater levels. Fair groundwater quality for irrigation was observed in the upstream and downstream wells. After excluding Mn and limiting wastewater leaching, the quality improved to good. Effective treatment is recommended before using groundwater for household and livestock purposes, as the GQI was marginal in both upstream and downstream areas for those uses. The HRA model indicated that dermal exposure to groundwater poses no carcinogenic or non-carcinogenic risks to adults or children. Nevertheless, limitations of the current work include examining only 20 wells once during barrage construction and focusing solely on the barrage's effects on the groundwater table, thereby excluding other stressors.

In the context of environmental implications, the combined use of groundwater-level analysis, groundwater-quality indices, and human health risk assessment provides a comprehensive framework. Overall, our study, based on extensive data, broadened our knowledge in that regard and showed that barrage construction raises groundwater levels in Assiut City and its surrounding areas, necessitating proper management to mitigate future environmental impacts. Our study underscores the need for further research to clarify manganese patterns in groundwater and assess potential risks. The present study provides a robust approach for depicting barrage effects and refining mitigation strategies to support better decision-making.

## Supplementary Information

Below is the link to the electronic supplementary material.

Supplementary file1 (DOCX 2856 KB)

## Data Availability

All data supporting the findings of this study are available in the paper and its Supplementary Information. Data will be available on reasonable request.
