# Supplementary material for: Environmental impact of the barrage construction on groundwater
Source: Environ Geochem Health. 2026 May 19;48(8):358. doi: 10.1007/s10653-026-03232-6 (PMC13186817; doi:10.1007/s10653-026-03232-6)
Supplement: Supplementary file 1 — Supplementary file1 (DOCX 2856 KB) [file 10653_2026_3232_MOESM1_ESM.docx]

**Supporting Information**

**Environmental Impact of the Barrage Construction on Groundwater**

Sherif M. Aboueldahab^1, 2^, El-Montser M. Seleem^1^, Ahmed M. Orabi^1^, Salah A. M.

Zeid^1^, Mariam Metwaly^3^, Mahmoud A. Abdelhafiz^1*^

^1^Geology Department, Faculty of Science, Al-Azhar University, Assiut 71524, Egypt

^2^Environmental Quality Management, Egyptian Environmental Affairs Agency, Assiut, Egypt

^3^Conservation Department, Faculty of Archaeology, Zagazig University, 44519, Egypt

***Corresponding author**:

Mahmoud Abdelkarim Abdelhafiz

ORCID: 0000-0001-7640-7067

Phone: +20-1066094214

Email: [elkarim_mahmoud@azhar.edu.eg](mailto:elkarim_mahmoud@azhar.edu.eg) ; [abdelhafizma@gmail.com](mailto:abdelhafizma@gmail.com)

**Supporting information contains:**

13 Pages, 1 Figure, 7 Tables

**Text S1.** **Groundwater quality index model calculation**

As it is an effective tool for evaluating water quality, the groundwater quality index (GQI) was calculated using the CWQI program (CCME 2017) according to the following:

$$GQI=100-\left( \frac{\sqrt{{F_{1}}^{2}+{F_{2}}^{2}+{F_{3}}^{2}}}{1.732} \right)$$

Where the divisor 1.732 normalizes the values to a 0-100 scale, where 0 denotes the "worst" groundwater quality, and 100 indicates the "best" groundwater quality.F_1_, F_2_, and F_3_ represent scope, frequency, and amplitude, respectively. These three factors are calculated as follows:

$$F_{1}={Number of failed variables}/{Total number of variables}\times100$$

$$F_{2}={Number of failed tests}/{Total number of tests}\times100$$

$$F_{3}={nse}/{0.01nse + 0.01}$$

$$nse={\sum_{i=1}^{n} excrsion \left( i \right)}/{Total number of tests}$$

$$xcursion \left( \boldsymbol{i} \right)={Failed test value}/{Objective}-1$$

**Text S2. Health risk assessment model calculation**

Dermal exposure to heavy metals is considered, given that groundwater is used extensively for irrigation, to assess human health risk. The dermal HRA (HRA*_d_*) model is calculated as follows:

$PDD=\frac{C\times SA\times ABS\times CF\times ET\times KP\times EV\times ED\times EF}{BW\times AT}$

$$HQ={PDD}/{{RfD}_{d}}$$

$${RfD}_{d}={RfD}_{i}\times{ABS}_{Gi}$$

$$HI=\sum HQ=\sum{PDD}/{RfD}$$

$$CR=PDD\times{CSF}_{d}$$

$${CSF}_{d}={{CSF}_{i}}/{{ABS}_{Gi}}$$

$${CR}_{T}=\sum CR=\sum PDD \times CSF$$

Where PDD represents the probable daily dose of exposure; C is the concentration of metal in water (mg/L); SA is the skin surface area exposed to water (6032 cm^2^ for adults; 2373 cm^2^ for children); ABS is the absorption ratio of dermal contact (0.001); CF is conversion factor (0.001); ET is contact duration (0.71 h d^−1^ for adults; 0.54 h d^−1^ for children); KP is the coefﬁcient of skin permeability (0.001 cm h^−1^); EV is daily exposure frequency of dermal contact (1), ED is exposure duration (49 years for adults; 5 years for children), EF is exposure frequency (350 d), body weight (70 kg for adults; 20 kg for children) and average time (ED×365 for non-carcinogenic and lifetime (70)×365 for carcinogenic risk). The parameter values were taken from prior studies (Abdelhafiz et al. 2021, USEPA 2014, Zhou et al. 2020)

HQ is the hazard quotient which reflects non-carcinogenic risk of a single metal through dermal contact; RfD*_d_* and RfD*_i_* are toxicity reference dose in mg kg^−1^ d^−1^ for non-carcinogenic pollutants through dermal and oral intake exposure pathway, respectively; ABS_Gi_ is the gastrointestinal absorption factor; CR is carcinogenic risk; CSF_d_ and CSF_i_ is the cancer slope factor of carcinogenic risk through the dermal contact and oral intake and exposure pathway, respectively; HI and CR_T_ are hazard index and total carcinogenic risk for evaluate the non-carcinogenic and carcinogenic risks from multiple metals, respectively. The carcenigenic and non-carcinogenic parameters valuves are calculated and given from USEPA (2019), Abdelhafiz et al. (2021), and Shomar andRashkeev (2021).


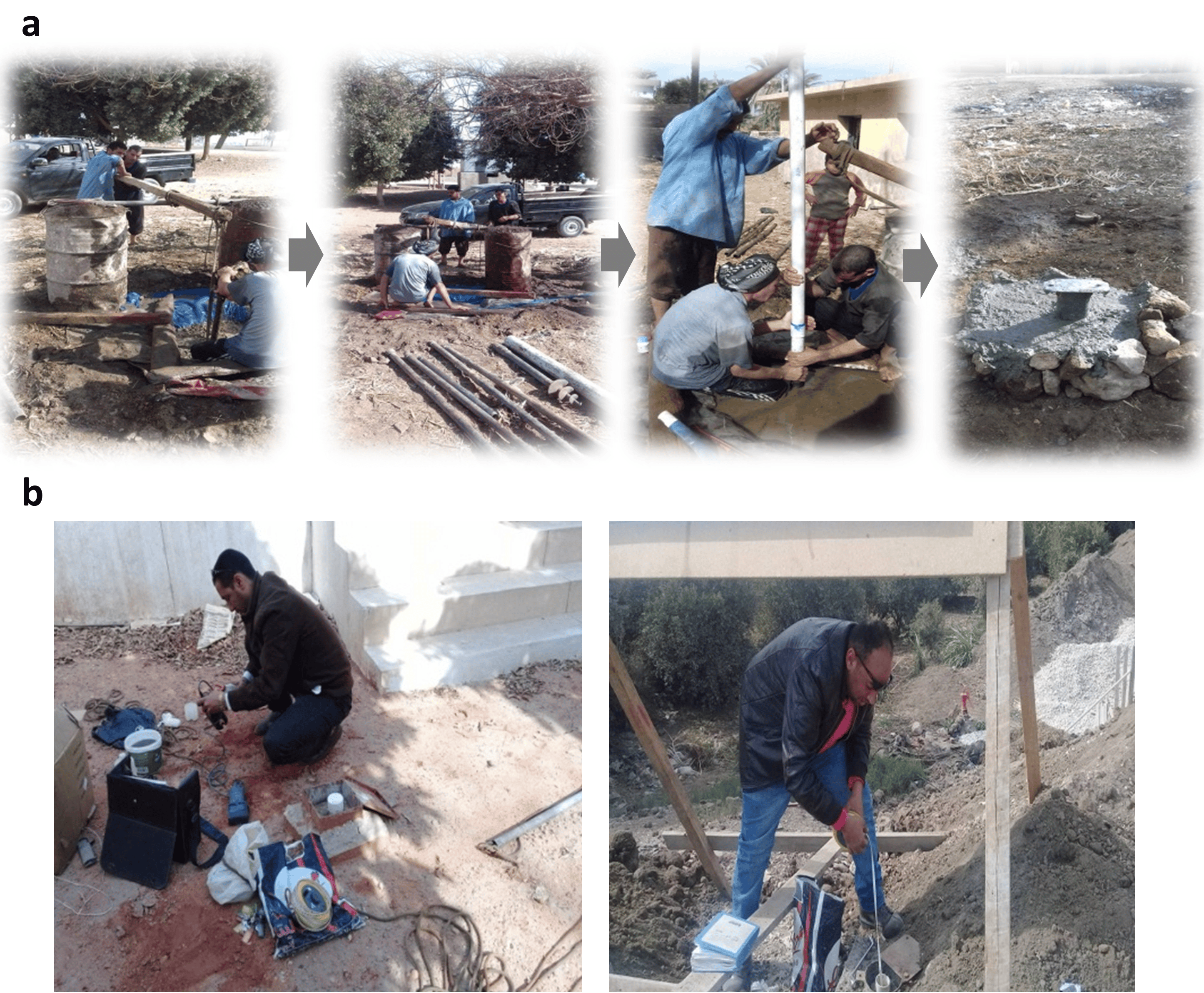


**Figure S1** Drilling procedures of observation wells for monitoring the groundwater levels (a), and measuring groundwater levels and collecting samples from observation wells (b).

**Table S1** Sampling locations and well names

| Sample No. | | Well name | X | Y |
| --- | --- | --- | --- | --- |
| 1 | Upstream | Tal Zayed - EL Badari | 31.41819527 | 26.96446391 |
| 2 |  | EL Shamia - Sahel Selim | 31.35162871 | 27.06408144 |
| 3 |  | Awlad Ibrahim | 31.24906695 | 27.15484606 |
| 4 |  | EL Matea | 31.29674283 | 27.13989299 |
| 5 |  | Magris - Sidfa | 31.37825059 | 26.97852974 |
| 6 |  | EL Huraika - Tama | 31.39173022 | 26.86480956 |
| 7 |  | Nagaa Hamad - Tahta | 31.45633261 | 26.81377811 |
| 8 |  | EL Ezba EL Mostagadh - Tama | 31.45288179 | 26.8577855 |
| 9 | Downstream | El Helaly St., Assiut | 31.19319309 | 27.18380572 |
| 10 |  | (Tagneed) Beside Recruitment | 31.18465508 | 27.18931435 |
| 11 |  | Beside Teraet El Ibrahimeya | 31.18690438 | 27.19694581 |
| 12 |  | (Welydia Kornish) Alwaleediyah 1 | 31.18447985 | 27.20254063 |
| 13 |  | Ezbet Khalaf village | 31.15087941 | 27.19305697 |
| 14 |  | El Arbaen, Assiut | 31.18549748 | 27.16338992 |
| 15 |  | Upper Bridge ELWasta | 31.22085247 | 27.18768007 |
| 16 |  | El Maasara Village-El Fath District | 31.2060649 | 27.20029491 |
| 17 |  | Al Asara Village - Al Fath Center | 31.200595 | 27.20496202 |
| 18 |  | ELWasta | 31.20425376 | 27.18995644 |
| 19 |  | Qanatir location | 31.23518478 | 27.25588306 |
| 20 |  | Alwaleediyah 2 - Taftesh El Ray | 31.1824433 | 27.19997009 |

**Table S2** Measured parameters and analytical method according to APHA (2017).

| Item | Abbreviations | Units | Reference method | Analytical method |
| --- | --- | --- | --- | --- |
| pH | pH | pH unit | SM 4500-H^+^B | pH-meter |
| Electrical conductivity | EC | µs/cm | SM 2510 B | Electrometric |
| Dissolved Oxygen | DO | mg/L | SM 4500 – O-G | Membrane electrode method |
| Nitrate nitrogen | NO_3_^¯^ | mg/L | SM 4500 – (NO_3_^¯^ - N)B | UV spectrophotometer |
| Ammonia | NH_4_^+^ | mg/L | SM 4500 – (NH_4_ – N) F | Phonate method |
| Total suspended solids | TSS | mg/L | SM-2540 D | EDTA Micrometric |
| Biological oxygen demand | BOD^2+^ | mg/L | 5210B | 5 – Day BOD test |

**Table S3** Irrigation groundwater salinity categories based on TDS and EC according to EPA (2024), FAO (1985), and Richards (1954).

| Classes | Categories | TDS (mg/L) | Samples % | EC (µS/cm) | Samples % |
| --- | --- | --- | --- | --- | --- |
| I | Excellent | < 175 | -- | < 250 | -- |
| II | Good | 175 – 525 | 20 | 250 – 750 | 15 |
| III | Permissible | 525 – 1400 | 75 | 750 – 2000 | 85 |
| IV | Doubtful | 1400 – 2100 | 5 | 2000 – 3000 | -- |
| V | Unsuitable | > 2100 | -- | > 3000 | -- |

**Table S4** Hazard quotient as non-carcinogenic risks and total hazards (HI) through dermal exposure to contaminants in upstream groundwater for both adults and children

|  | **Adults** | |  |  |  |  |  |  |  | **Children** | |  |  |  |  |  |  |  |
| --- | --- | --- | --- | --- | --- | --- | --- | --- | --- | --- | --- | --- | --- | --- | --- | --- | --- | --- |
|  | Cd | Al | Cr | Cu | Fe | Mn | Ni | Zn | **HI** | Cd | Al | Cr | Cu | Fe | Mn | Ni | Zn | **HI** |
| **1** | 8.9E-08 | 4.2E-09 | 6.9E-09 | 1.5E-07 | 1.8E-07 | 1.6E-05 | 2.4E-08 | 1.7E-07 | 1.7E-05 | 9.3E-08 | 4.4E-09 | 7.2E-09 | 1.6E-07 | 1.9E-07 | 1.7E-05 | 2.5E-08 | 1.8E-07 | 1.8E-05 |
| **2** | 3.8E-08 | 5.9E-09 | 9.3E-09 | 1.0E-07 | 1.7E-07 | 2.9E-05 | 2.3E-08 | 3.3E-08 | 2.9E-05 | 3.9E-08 | 6.2E-09 | 9.7E-09 | 1.1E-07 | 1.8E-07 | 3.0E-05 | 2.5E-08 | 3.4E-08 | 3.0E-05 |
| **3** | 1.6E-07 | 1.1E-08 | 1.3E-08 | 1.1E-07 | 1.7E-06 | 4.8E-05 | 6.1E-09 | 2.7E-08 | 5.0E-05 | 1.7E-07 | 1.1E-08 | 1.4E-08 | 1.1E-07 | 1.8E-06 | 5.0E-05 | 6.4E-09 | 2.8E-08 | 5.3E-05 |
| **4** | 6.6E-08 | 8.6E-09 | 1.4E-08 | 6.2E-08 | 1.5E-07 | 6.7E-06 | 7.6E-09 | 2.1E-08 | 7.1E-06 | 6.9E-08 | 9.0E-09 | 1.4E-08 | 6.5E-08 | 1.6E-07 | 7.0E-06 | 7.9E-09 | 2.2E-08 | 7.4E-06 |
| **5** | 1.2E-07 | 8.0E-09 | 1.3E-08 | 2.0E-07 | 1.1E-06 | 5.0E-05 | 1.3E-08 | 2.0E-08 | 5.2E-05 | 1.3E-07 | 8.4E-09 | 1.4E-08 | 2.0E-07 | 1.1E-06 | 5.3E-05 | 1.4E-08 | 2.1E-08 | 5.4E-05 |
| **6** | 6.2E-07 | 8.6E-09 | 4.6E-09 | 3.0E-07 | 4.2E-07 | 1.1E-04 | 6.7E-09 | 6.9E-08 | 1.1E-04 | 6.5E-07 | 9.0E-09 | 4.8E-09 | 3.1E-07 | 4.4E-07 | 1.2E-04 | 7.0E-09 | 7.2E-08 | 1.2E-04 |
| **7** | 2.9E-07 | 8.3E-09 | 4.6E-09 | 1.4E-07 | 1.8E-07 | 3.2E-05 | 1.1E-08 | 2.8E-08 | 3.3E-05 | 3.0E-07 | 8.7E-09 | 4.8E-09 | 1.5E-07 | 1.9E-07 | 3.4E-05 | 1.2E-08 | 2.9E-08 | 3.5E-05 |
| **8** | 9.9E-07 | 8.6E-09 | 1.3E-08 | 2.3E-07 | 4.4E-07 | 7.4E-05 | 8.5E-09 | 7.0E-08 | 7.5E-05 | 1.0E-06 | 9.0E-09 | 1.4E-08 | 2.4E-07 | 4.6E-07 | 7.7E-05 | 8.9E-09 | 7.4E-08 | 7.9E-05 |
| **Mean** | **3.0E-07** | **7.9E-09** | **9.8E-09** | **1.6E-07** | **5.4E-07** | **4.6E-05** | **1.3E-08** | **5.5E-08** | **4.7E-05** | **3.1E-07** | **8.2E-09** | **1.0E-08** | **1.7E-07** | **5.7E-07** | **4.8E-05** | **1.3E-08** | **5.8E-08** | **4.9E-05** |
| **Min** | **3.8E-08** | **4.2E-09** | **4.6E-09** | **6.2E-08** | **1.5E-07** | **6.7E-06** | **6.1E-09** | **2.0E-08** | **7.1E-06** | **3.9E-08** | **4.4E-09** | **4.8E-09** | **6.5E-08** | **1.6E-07** | **7.0E-06** | **6.4E-09** | **2.1E-08** | **7.4E-06** |
| **Max** | **9.9E-07** | **1.1E-08** | **1.4E-08** | **3.0E-07** | **1.7E-06** | **1.1E-04** | **2.4E-08** | **1.7E-07** | **1.1E-04** | **1.0E-06** | **1.1E-08** | **1.4E-08** | **3.1E-07** | **1.8E-06** | **1.2E-04** | **2.5E-08** | **1.8E-07** | **1.2E-04** |

**Table S5** Hazard quotient as non-carcinogenic risks and total hazards (HI) through dermal exposure to contaminants in downstream groundwater for both adults and children

|  | **Adults** | |  |  |  |  |  |  |  | **Children** | |  |  |  |  |  |  |  |
| --- | --- | --- | --- | --- | --- | --- | --- | --- | --- | --- | --- | --- | --- | --- | --- | --- | --- | --- |
|  | Cd | Al | Cr | Cu | Fe | Mn | Ni | Zn | **HI** | Cd | Al | Cr | Cu | Fe | Mn | Ni | Zn | **HI** |
| **9** | 6.5E-07 | 8.4E-09 | 1.1E-08 | 1.1E-07 | 2.4E-07 | 1.5E-05 | 9.4E-09 | 1.6E-08 | 1.6E-05 | 6.8E-07 | 8.8E-09 | 1.2E-08 | 1.1E-07 | 2.5E-07 | 1.6E-05 | 9.9E-09 | 1.6E-08 | 1.7E-05 |
| **10** | 7.7E-07 | 8.6E-09 | 1.5E-08 | 8.8E-08 | 1.2E-07 | 1.3E-05 | 4.9E-09 | 3.5E-08 | 1.4E-05 | 8.0E-07 | 9.0E-09 | 1.6E-08 | 9.2E-08 | 1.2E-07 | 1.4E-05 | 5.2E-09 | 3.6E-08 | 1.5E-05 |
| **11** | 5.9E-07 | 8.6E-09 | 1.1E-08 | 1.1E-07 | 4.3E-07 | 4.7E-05 | 3.9E-09 | 1.3E-08 | 4.8E-05 | 6.1E-07 | 9.0E-09 | 1.2E-08 | 1.2E-07 | 4.5E-07 | 4.9E-05 | 4.1E-09 | 1.3E-08 | 5.0E-05 |
| **12** | 7.5E-07 | 1.1E-08 | 1.2E-08 | 1.4E-07 | 2.2E-07 | 5.0E-05 | 9.4E-09 | 4.6E-08 | 5.2E-05 | 7.8E-07 | 1.1E-08 | 1.3E-08 | 1.5E-07 | 2.3E-07 | 5.3E-05 | 9.9E-09 | 4.8E-08 | 5.4E-05 |
| **13** | 1.2E-06 | 1.0E-08 | 7.2E-09 | 1.6E-07 | 3.2E-07 | 3.8E-05 | 5.6E-09 | 5.9E-08 | 4.0E-05 | 1.3E-06 | 1.1E-08 | 7.5E-09 | 1.7E-07 | 3.3E-07 | 4.0E-05 | 5.9E-09 | 6.2E-08 | 4.2E-05 |
| **14** | 3.8E-07 | 7.5E-09 | 7.1E-09 | 8.1E-08 | 9.4E-08 | 9.5E-06 | 6.7E-09 | 4.6E-08 | 1.0E-05 | 3.9E-07 | 7.9E-09 | 7.4E-09 | 8.4E-08 | 9.8E-08 | 1.0E-05 | 7.0E-09 | 4.9E-08 | 1.1E-05 |
| **15** | 7.8E-06 | 4.5E-09 | 2.4E-09 | 3.4E-07 | 2.5E-06 | 5.9E-04 | 5.8E-09 | 6.8E-07 | 6.0E-04 | 8.1E-06 | 4.7E-09 | 2.5E-09 | 3.5E-07 | 2.6E-06 | 6.2E-04 | 6.1E-09 | 7.2E-07 | 6.3E-04 |
| **16** | 6.2E-07 | 1.1E-08 | 3.1E-08 | 8.8E-08 | 2.2E-07 | 4.8E-05 | 8.8E-08 | 1.1E-08 | 4.9E-05 | 6.5E-07 | 1.1E-08 | 3.2E-08 | 9.2E-08 | 2.3E-07 | 5.0E-05 | 9.2E-08 | 1.2E-08 | 5.1E-05 |
| **17** | 3.6E-07 | 8.4E-09 | 2.6E-09 | 1.2E-07 | 1.1E-07 | 4.4E-05 | 3.9E-09 | 5.7E-08 | 4.4E-05 | 3.7E-07 | 8.8E-09 | 2.7E-09 | 1.3E-07 | 1.2E-07 | 4.6E-05 | 4.1E-09 | 6.0E-08 | 4.6E-05 |
| **18** | 8.3E-07 | 8.4E-09 | 4.1E-09 | 1.6E-07 | 1.5E-06 | 9.1E-05 | 3.1E-09 | 3.8E-08 | 9.3E-05 | 8.7E-07 | 8.8E-09 | 4.3E-09 | 1.7E-07 | 1.6E-06 | 9.5E-05 | 3.3E-09 | 4.0E-08 | 9.8E-05 |
| **19** | 1.3E-06 | 1.1E-08 | 7.3E-09 | 1.2E-07 | 6.0E-07 | 1.1E-04 | 1.3E-08 | 5.4E-08 | 1.1E-04 | 1.3E-06 | 1.1E-08 | 7.7E-09 | 1.2E-07 | 6.2E-07 | 1.1E-04 | 1.4E-08 | 5.6E-08 | 1.2E-04 |
| **20** | 1.9E-06 | 1.1E-08 | 1.0E-08 | 1.6E-07 | 7.6E-07 | 4.0E-05 | 8.6E-09 | 2.6E-08 | 4.3E-05 | 2.0E-06 | 1.1E-08 | 1.0E-08 | 1.6E-07 | 8.0E-07 | 4.2E-05 | 9.0E-09 | 2.7E-08 | 4.5E-05 |
| **Mean** | **1.4E-06** | **9.0E-09** | **1.0E-08** | **1.4E-07** | **5.9E-07** | **9.1E-05** | **1.4E-08** | **9.0E-08** | **9.3E-05** | **1.5E-06** | **9.4E-09** | **1.1E-08** | **1.5E-07** | **6.2E-07** | **9.5E-05** | **1.4E-08** | **9.5E-08** | **9.8E-05** |
| **Min** | **3.6E-07** | **4.5E-09** | **2.4E-09** | **8.1E-08** | **9.4E-08** | **9.5E-06** | **3.1E-09** | **1.1E-08** | **1.0E-05** | **3.7E-07** | **4.7E-09** | **2.5E-09** | **8.4E-08** | **9.8E-08** | **1.0E-05** | **3.3E-09** | **1.2E-08** | **1.1E-05** |
| **Max** | **7.8E-06** | **1.1E-08** | **3.1E-08** | **3.4E-07** | **2.5E-06** | **5.9E-04** | **8.8E-08** | **6.8E-07** | **6.0E-04** | **8.1E-06** | **1.1E-08** | **3.2E-08** | **3.5E-07** | **2.6E-06** | **6.2E-04** | **9.2E-08** | **7.2E-07** | **6.3E-04** |

**Table S6** Carcinogenic and total carcinogenic risk results of contaminants in upstream groundwater through dermal contact for adults and children

| Well No. | **Adults** | |  |  | **Children** | |  |  |
| --- | --- | --- | --- | --- | --- | --- | --- | --- |
|  | Cd | Cr | Ni | **CR_T_** | Cd | Cr | Ni | **CR_T_** |
| **1** | 1.19E-11 | 2.97E-08 | 3.07E-10 | 3.00E-08 | 1.27E-12 | 3.17E-09 | 3.28E-11 | 3.20E-09 |
| **2** | 4.99E-12 | 3.99E-08 | 2.99E-10 | 4.02E-08 | 5.34E-13 | 4.26E-09 | 3.19E-11 | 4.30E-09 |
| **3** | 2.12E-11 | 5.75E-08 | 7.81E-11 | 5.76E-08 | 2.27E-12 | 6.15E-09 | 8.35E-12 | 6.16E-09 |
| **4** | 8.74E-12 | 5.89E-08 | 9.64E-11 | 5.90E-08 | 9.34E-13 | 6.30E-09 | 1.03E-11 | 6.31E-09 |
| **5** | 1.62E-11 | 5.74E-08 | 1.70E-10 | 5.76E-08 | 1.73E-12 | 6.13E-09 | 1.82E-11 | 6.15E-09 |
| **6** | 8.24E-11 | 1.97E-08 | 8.52E-11 | 1.99E-08 | 8.80E-12 | 2.10E-09 | 9.11E-12 | 2.12E-09 |
| **7** | 3.87E-11 | 1.97E-08 | 1.44E-10 | 1.99E-08 | 4.14E-12 | 2.10E-09 | 1.54E-11 | 2.12E-09 |
| **8** | 1.31E-10 | 5.56E-08 | 1.08E-10 | 5.58E-08 | 1.40E-11 | 5.94E-09 | 1.15E-11 | 5.96E-09 |
| **Mean** | 3.94E-11 | 4.23E-08 | 1.61E-10 | 4.25E-08 | 4.21E-12 | 4.52E-09 | 1.72E-11 | 4.54E-09 |
| **Min** | 4.99E-12 | 1.97E-08 | 7.81E-11 | 1.99E-08 | 5.34E-13 | 2.10E-09 | 8.35E-12 | 2.12E-09 |
| **Max** | 1.31E-10 | 5.89E-08 | 3.07E-10 | 5.90E-08 | 1.40E-11 | 6.30E-09 | 3.28E-11 | 6.31E-09 |

**Table S7** Carcinogenic and total carcinogenic risk results of contaminants in downstream groundwater through dermal contact for adults and children

| Well No. | **Adults** | |  |  | **Children** | |  |  |
| --- | --- | --- | --- | --- | --- | --- | --- | --- |
|  | Cd | Cr | Ni | **CR_T_** | Cd | Cr | Ni | **CR_T_** |
| **9** | 8.61E-11 | 4.84E-08 | 1.20E-10 | 4.86E-08 | 9.21E-12 | 5.18E-09 | 1.28E-11 | 5.20E-09 |
| **10** | 1.02E-10 | 6.44E-08 | 6.28E-11 | 6.45E-08 | 1.09E-11 | 6.88E-09 | 6.71E-12 | 6.90E-09 |
| **11** | 7.80E-11 | 4.90E-08 | 5.01E-11 | 4.91E-08 | 8.34E-12 | 5.23E-09 | 5.35E-12 | 5.25E-09 |
| **12** | 9.93E-11 | 5.22E-08 | 1.20E-10 | 5.24E-08 | 1.06E-11 | 5.58E-09 | 1.28E-11 | 5.60E-09 |
| **13** | 1.66E-10 | 3.10E-08 | 7.14E-11 | 3.12E-08 | 1.77E-11 | 3.31E-09 | 7.63E-12 | 3.33E-09 |
| **14** | 4.99E-11 | 3.06E-08 | 8.56E-11 | 3.07E-08 | 5.34E-12 | 3.27E-09 | 9.15E-12 | 3.28E-09 |
| **15** | 1.03E-09 | 1.04E-08 | 7.40E-11 | 1.15E-08 | 1.11E-10 | 1.11E-09 | 7.91E-12 | 1.23E-09 |
| **16** | 8.30E-11 | 1.33E-07 | 1.12E-09 | 1.34E-07 | 8.87E-12 | 1.42E-08 | 1.20E-10 | 1.44E-08 |
| **17** | 4.74E-11 | 1.13E-08 | 4.97E-11 | 1.14E-08 | 5.07E-12 | 1.20E-09 | 5.31E-12 | 1.21E-09 |
| **18** | 1.10E-10 | 1.76E-08 | 3.96E-11 | 1.78E-08 | 1.17E-11 | 1.88E-09 | 4.23E-12 | 1.90E-09 |
| **19** | 1.67E-10 | 3.16E-08 | 1.64E-10 | 3.19E-08 | 1.79E-11 | 3.38E-09 | 1.76E-11 | 3.41E-09 |
| **20** | 2.55E-10 | 4.31E-08 | 1.09E-10 | 4.35E-08 | 2.73E-11 | 4.61E-09 | 1.17E-11 | 4.65E-09 |
| **Mean** | 1.90E-10 | 4.36E-08 | 1.73E-10 | 4.39E-08 | 2.03E-11 | 4.65E-09 | 1.84E-11 | 4.69E-09 |
| **Min** | 4.74E-11 | 1.04E-08 | 3.96E-11 | 1.14E-08 | 5.07E-12 | 1.11E-09 | 4.23E-12 | 1.21E-09 |
| **Max** | 1.03E-09 | 1.33E-07 | 1.12E-09 | 1.34E-07 | 1.11E-10 | 1.42E-08 | 1.20E-10 | 1.44E-08 |

**References**

Abdelhafiz MA, Elnazer AA, Seleem E-MM, Mostafa A, Al-Gamal AG, Salman SA, Feng X (2021): Chemical and bacterial quality monitoring of the Nile River water and associated health risks in Qena–Sohag sector, Egypt. Environmental Geochemistry and Health 43, 4089-4104. <https://doi.org/10.1007/s10653-021-00893-3>

APHA (2017): Standard methods for the examination of water and wastewater, 21st edn. American Public Health Association/American Water Works Association/Water Environment Federation, Washington, DC

Ayers R, Westcot D (1985): Water quality for agriculture, irrigation and drainage. Paper No. 29. Food and Agriculture Organization (FAO) of the United Nations, Rome, Italy, p 1–117

CCME 2017: CCME Water Quality Index 2.0 User’s Manual – 2017 update, Canadian Council of Ministers of the Environment

EPA 2024: What's in My Water? Texas Cooperative Extension. Texas A&M University System. E-176, 4-03

Richards LA (1954): Diagnosis and improvement of saline and alkali soils. Agricultural hand book 60. U.S. Dept. of Agriculture, Washington D.C., 160 p.

Shomar B, Rashkeev SN (2021): A comprehensive risk assessment of toxic elements in international brands of face foundation powders. Environmental Research 192, 110274. <https://doi.org/10.1016/j.envres.2020.110274>

USEPA 2014: Human Health Evaluation Manual, Supplemental Guidance: Update of Standard Default Exposure Factors. (Publication 9200.1-120). , Washington, D.C.: Office of Solid Waste and Emergency Response, U.S. EPA.

USEPA (2019): Guidelines for Human Exposure Assessment., (EPA/100/B-19/001). U.S. Environmental Protection Agency Washington, D.C.: Risk Assessment Forum, U.S. EPA.

Zhou Y, Li P, Chen M, Dong Z, Lu C (2020): Groundwater quality for potable and irrigation uses and associated health risk in southern part of Gu’an County, North China Plain. Environmental Geochemistry and Health. <https://doi.org/10.1007/s10653-020-00553-y>
